# Self-supervised graph neural network with pre-training generative learning for recommendation systems

**DOI:** 10.1038/s41598-022-19528-3

**Published:** 2022-09-23

**Authors:** Xin Min, Wei Li, Jinzhao Yang, Weidong Xie, Dazhe Zhao

**Affiliations:** 1 School of Computer Science and Engineering, Northeastern University, Shenyang, 110000 China; 2 Key Laboratory of Intelligent Computing in Medical Image (MIIC), Northeastern University, Shenyang, 110000 China

**Keywords:** Environmental social sciences, Engineering

## Abstract

The case assignment system is an essential system of case management and assignment within the procuratorate and is an important aspect of judicial fairness and efficiency. However, existing methods mostly use manual or random case assignment, which leads to unbalanced case distribution. Moreover, the relationship between prosecutors and case categories usually shows a power-law distribution in real-world data. Therefore, in this paper, we describe the case rationality assignment as a recommendation problem under the power-law distributed data. To solve the above problems, we propose an end-to-end Self-supervised Graph neural network model with Pre-training Generative learning for Recommendation (SGPGRec), the main idea of which is to capture self-supervised signals using intra-node features and inter-node correlations in the data, and generate the data representation by pre-training to improve the recommendation results. To be specific, we designed three auxiliary self-supervised tasks based on the prosecutor-case category interaction graph and the data distribution to obtain feature representations of prosecutors, case categories, and the interaction information between them. Then we constructed an end-to-end graph neural network recommendation model by the interaction information based on the data characteristics of the power-law distribution. Finally, extensive experimental consistency on a real-world dataset from three procuratorates shows that our method is effective compared to several yet competing baseline methods and further supports the development of an intelligent case assignment system with adequate performance.

## Introduction

With the rapid development of the Internet, the application of artificial intelligence in the judicial field is becoming more widespread, which can significantly improve the efficiency of handling cases. In practice, Case assignment directly affects the efficiency of case processing, prosecutors’ competence development, and the improvement of the incentive system. Therefore, it is vital to develop a compelling case assignment method based on the information of the case and the prosecutor’s ability.

Currently, the procuratorate has established the pattern of “random case assignment as the main mode and designated case assignment as a supplement.” This pattern refers to the case assignment based on manual designation or specific rules. The advantage of the pattern is that the case manager can give full play to their flexibility in assigning cases to prosecutors and making optimal assignments. However, there are problems such as excessive human factors, lack of objectivity, and lack of openness in the case assignment process. Moreover, this pattern cannot flexibly identify the specific situation of the case to select the best contractor prosecutor with the prosecutor’s ability and the case’s complexity.

If we represent prosecutors/cases as a collection of nodes in the data, then case assignment can be described as a similar item-user recommendation problem. Learning high-quality user and item representations from interaction data is the core problem of recommendation. Traditional methods such as collaborative filtering^1–3^ enriched the interaction information to learn better representations. But these traditional methods share the same problem: items with high interaction have a higher impact on representational learning, making the model biased towards recommending head items. Recently, graph neural networks can perform representation learning for higher-order connectivity in user-item graphs. It integrates multi-level neighbors into node representation learning to achieve the best recommendation^4,5^. The prosecutor and case category usually show a power-law distribution in the judicial scenario. The rationality of case assignment refers to assigning cases to prosecutors reasonably and discovering potential case-prosecutor relationships. Hence, the recommendation model needs to consider the distribution of case categories.

In this paper, we propose an end-to-end graph neural network recommendation model based on self-supervised learning to use case feature and prosecutor ability feature fully. The main idea of this method is to capture self-supervised signals with intra-node features and inter-node correlations in the graph and generate the node representation by pre-training tasks to improve the recommendation results. Specifically, we first designed three auxiliary self-supervised tasks based on the prosecutor-case category interaction graph to obtain the representations of prosecutors, case categories, and the interaction between them. Then we constructed an end-to-end graph neural network recommendation model with the attention mechanism based on the data distribution. Finally, we tested and integrated on a real dataset to verify the validity and usefulness of the model.

In summary, this paper has the following contributions.

- This paper proposes a self-supervised end-to-end graph neural network recommendation model SGCRec, pre-trained with generated intra-node features and inter-node correlations to provide auxiliary signals for representation learning.
- We propose three practical pre-training generative tasks: prosecutor portrait depiction, case workload prediction, and case feature selection, which can learn head and tail information under power-law distributed data in a balanced way.
- We conducted extensive experiments on a real-world dataset. These experiments validate the soundness and robustness of our recommendation model and provide some new ideas for the recommendation problem in the judicial domain.

The rest of the paper is organized as follows: Section "Related work" presents the related work. "Proposed method" section presents our proposed method. Section "Experiment" performs the experimental evaluation. Section "Analysis of experimental results"results analysis of our method. Finally, section " Conclusion" summarizes our work.

## Related work

### Graph neural recommendation models

In recent years, the development of graph neural networks has provided a strong foundation and opportunity for developing graph-related hybrid recommendation tasks^6,7^. In particular, GCN^8–10^, a general formulation of GNNs, is a first-order approximation to spectral graph convolution and has driven many graph neural recommendation models, such as GCMC^5^, NGCF^11^, and LightGCN^4^. These GCN-based graph neural networks all adopted embedding propagation to iteratively aggregate neighborhood embeddings. By stacking the propagation layers, each node can access the embeddings of higher-order neighbors^12^, instead of only first-order neighbors as in the traditional methods [MF, NCF]^1^^13^. Therefore GNN-based methods are the latest approaches in recommender systems with their advantages of handling structural and structural data.

### Self-supervised learning in recommender systems

Self-supervised learning focuses on mining its supervised signals from large-scale unsupervised data using an auxiliary task (pretext). Then, it trains the model with such constructed supervised signs to learn valuable representations for downstream tasks. The mainstream self-supervised learning includes three main categories: generative, contrastive, and adversarial, all of which have a wide range of applications^14^. In visual representation learning, several self-supervised targets are introduced to guide visual feature learning using intrinsically relevant supervised signals^15,16^. In natural language processing, the model learns to predict the next word or sentence based on the sequence above^17,18^. In addition, self-supervised learning is also widely used in graph data learning^19–21^. For example, InfoGraph^22^ and DGI^23^ learn node representations based on mutual information between nodes and local structures.

Inspired by the results of self-supervised learning in graph learning, some recent studies^24–26^ have transposed it to the recommendation scenario. S3-Rec^25^ tried to use the principle of mutual information maximization to perform sequence recommendations. In addition, Wu et al.^26^ summarized all the stochastic enhancements on the graph and unified them into a general self-supervised graph learning framework for the recommendation.

In contrast to the above methods, our work is the first to consider both intra-node features and inter-node correlations generated through pre-training tasks. In power-law distributed data, these generative signals with the attention mechanism can motivate the model to consider both head and long-tail information to improve the recommendation performance.

## Proposed method

**Figure 1:**
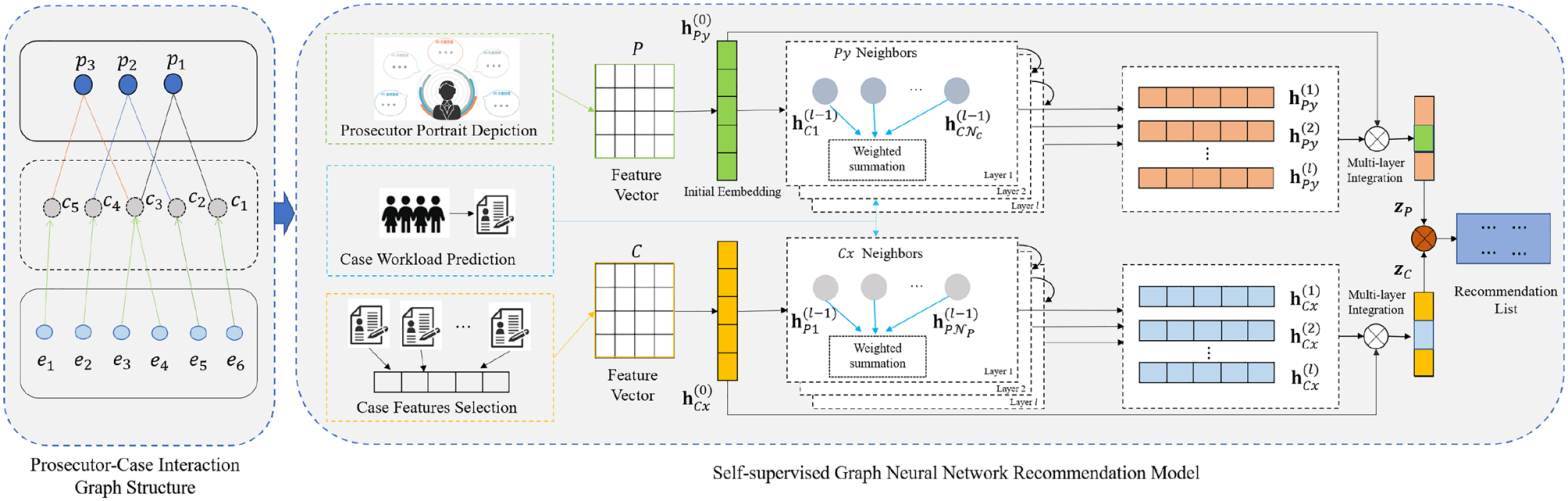
Overview of the proposed SGPGRec framework.

In this section, we present our method that utilizes intra-node features and inter-node correlations to capture self-supervised signals and generate the data representation by pre-training tasks to improve the recommendation results. The overall structure of the model is shown in Fig. 1. First, we introduce the relevant notation and graph structure. Then, we describe how the model is trained with three pre-training tasks. Finally, we present the self-supervised graph neural network recommendation process.

### Notations and graph structure

**Figure 2:**
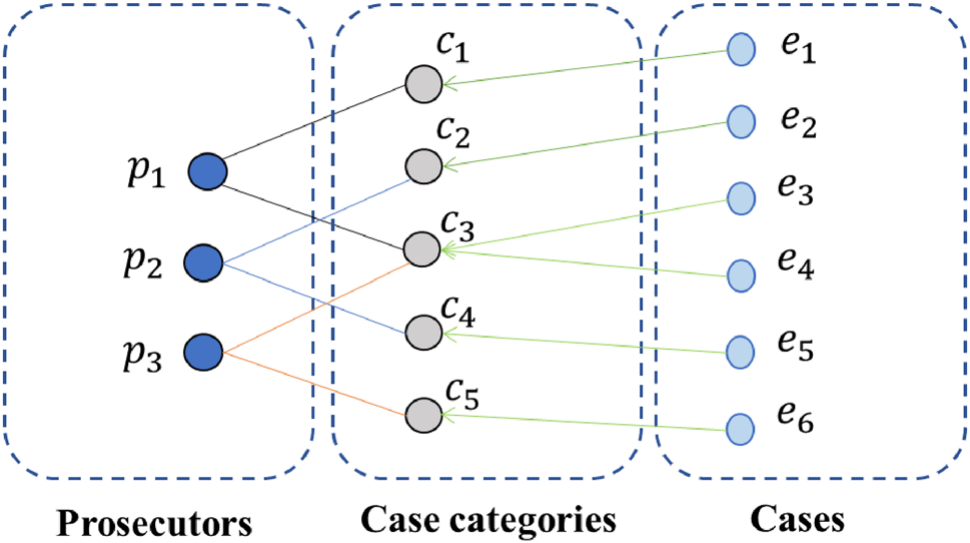
Prosecutor-case category interaction graph structure.

First, in the judicial scenario, we use the symbolic representation of complex information in case assignment data. Based on the interaction among prosecutors and cases, case types, we construct a heterogeneous graph $\mathcal {G}=(\mathcal {V}, \mathcal {E})$, as shown in Fig. 2. There are three kinds of nodes in the graph: prosecutor $P=\left\{ p_{1}, p_{2}, \cdots , p_{M}\right\}$, case category $C=\left\{ c_{1}, c_{2}, \cdots , c_{N}\right\}$, and case $E=\left\{ e_{1}, e_{2}, \cdots , e_{S}\right\}$ ,i.e., $\mathcal {V}=P \cup C \cup E$ and the set of edges $\mathcal {E}$ represents the relationship among prosecutor, case category, and case. Moreover, case, case category and prosecutor contains multiple features, and we denote case features by $\mathcal {A}_{E}=\left\{ a_{1}^{e}, \ldots , a_{m}^{e}\right\}$, case category features by $\mathcal {X}_{C}=\left\{ x_{1}^{c}, \ldots , x_{m}^{c}\right\}$ and prosecutor features by $\mathcal {B}_{P}=\left\{ b_{1}^{P}, \ldots , b_{n}^{P}\right\}$, where the case category features need to be learned from the case features. For example, cases have features such as the cause of referral, the number of people involved, and the amount of money involved, and prosecutors have features such as academic qualifications, the number of cases handled, and the quality of cases handled. We construct a heterogeneous graph by the interaction of multiple cases handled by each prosecutor, which is described in detail as follows.

We first categorize the cases according to the category so that prosecutors and case categories are connected in both directions in the graph, which model each prosecutor’s interaction with the case category. Since a case category contains multiple cases, a one-way edge from case to case category is created to message information from case information to case category only.

### Pre-training generative tasks

The traditional graph-based recommendation models^8,9^ usually employed a random feature representation of nodes to recommend the next item of the user, and the interaction between the user and the item were represented by the adjacency matrix only. However, those models of node-random initialization restricted the expressiveness of items and users, and the simple representation of interaction led to much information loss, so those model have some limitations in specific application scenarios. In judicial scenario, the prosecutor’s ability to handle the case, the complexity of the case, and the expected workload of the prosecutor in handling the case will affect the rationality of case assignment. Therefore, based on the previous study, we further integrate three pre-training tasks to enable the model to perform rational case assignment: prosecutor portrait depiction, case workload prediction, and case features selection. These three tasks are jointly optimized for the model.

#### Prosecutor portrait depiction

Prosecutor portrait depiction enables assessment of prosecutors’ ability to handle cases accurately. Therefore, we use the feature portrait depiction task to assess prosecutors’ case-handling ability based on the existing work^25,27^. For each prosecutor, the case-handling information provides detailed feature. Therefore, our goal is to enrich the prosecutor feature by portrait depiction.

For each prosecutor *P*, we first adopt the prosecutor’s historical case handling data to draw the prosecutors’ features in the dimensions of quantity, proportion and distribution, respectively. Then, we combine with the natural feature of prosecutors to normalize them. The profiles of prosecutors in each dimension are calculated as follows.

1 $$\begin{aligned} \mathcal {K}_{i}^{\prime }(P)=\alpha _{1} \sum _{q}^{Q} \llbracket b_{i}^{p}(q) \rrbracket +\alpha _{2} \sum _{r}^{R} \llbracket b_{i}^{p}(r) \rrbracket +\alpha _{3} \sum _{u}^{U} \llbracket b_{i}^{p}(u) \rrbracket \end{aligned}$$

where $\mathcal {K}_{i}^{\prime }(P)$ denotes the *i*-th dimensional feature drawing of prosecutor *P*, $\llbracket b_{i}^{p}(q) \rrbracket$ denotes normalized quantitative features, $\llbracket b_{i}^{p}(r) \rrbracket$ denotes normalized proportional features, and $\llbracket b_{i}^{p}(u) \rrbracket$ denotes normalized distributional features. $\alpha _{1}$, $\alpha _{2}$, and $\alpha _{3}$ denote the weights of the three indicator features.

For the prosecutors’ dimensional features, we adopt linear regression for prediction and a loss function with mean squared error (MSE) for optimization.

2 $$\begin{aligned} \mathcal {L}_{P P E}=\frac{1}{N} \sum _{n \in N}\left[ \mathcal {K}_{n}^{\prime }(P)-\mathcal {K}_{n}(P)\right] ^{2} \end{aligned}$$

#### Case workload prediction

Previous researches have indicated^27,28^ that adding more interaction to recommendations can significantly improve the performance of personalized recommendations. Because of the varying complexity of cases, the relationship between prosecutors and case categories measured only in terms of the number of handling cases would lose a great deal of critical interaction information. Therefore, we predict the expected case workload to generate prosecutor-case category correlation.

The expected workload for prosecutors *p* handling each case category can be expressed by the formula 3.

3 $$\begin{aligned} T_{p}^{m}=\frac{1}{S_{m}} \sum _{j \in S_{m}} W_{m} D t_{m}^{j} \end{aligned}$$

where $T_{p}^{m}$ denotes the expected workload of prosecutor *p* handing case category *m*. The $S_{m}$ denotes the total number of cases in case category *m*, The $W_{m}$ denotes the difficulty factor of case category *m*, and the $D t_{m}^{j}$ denotes the processing time of the *j*th case in case category *m*.

We formalize the task as a regression problem, so we use the Huber loss function as the loss function.

4 $$\begin{aligned} \mathcal {L}_{C W P}=\frac{1}{M} \sum _{m \in M} {\left\{ \begin{array}{ll}0.5\left( T_{p}^{m}-T_{p}^{c}\right) ^{2} &{} \text{ if } |T_{p}^{m}-T_{p}^{c}|<1 \\ |T_{p}^{m}-T_{p}^{c}|-0.5 &{} \text{ otherwise } \end{array}\right. } \end{aligned}$$

where $T_{p}^{m}$ and $T_{p}^{c}$ denote the predicted expected workload and the true expected workload, respectively.

#### Case features selection

Case feature selection enables the analysis of case complexity and generates case feature representation. For each case, the complexity of the case affects the difficulty of handling the case. Therefore, we aim to enrich the case feature representation by modelling the case complexity analysis model. We propose to model case complexity by feature selection methods, inspired by generative item feature learning methods such as^25,29^.

We adopt the GBDT^30^ for feature selection. We initially input the complexity labels of the cases and then obtain the complexity labels of the predicted cases by Eq. 5.

5 $$\begin{aligned} \left[ \mathcal {Y}^{\prime },\mathcal {A}_{c}{ }^{\prime }\right] \Leftarrow G B D T\left[ \mathcal {Y}, \mathcal {A}_{c}\right] , \mathcal {A}_{c}=\left\{ a_{1}^{c}, \ldots , a_{m}^{c}\right\} \end{aligned}$$

Where $\left[ \mathcal {Y}, \mathcal {A}_{c}\right]$ denotes the input case complexity labels and case features, respectively. In practice, there are two categories of case complexity labels, significant and ordinary. The $\left[ \mathcal {Y}^{\prime },\mathcal {A}_{c}{ }^{\prime }\right]$ denotes the classification result and the selected case features, respectively.

We formalize the feature selection task with average cross-entropy as the loss function.

6 $$\begin{aligned} \mathcal {L}_{C F S}=\frac{1}{M} \sum _{e \in {S}_{C}}-\log P\left( \mathcal {Y}_{e}^{\prime }=\mathcal {Y}_{e}\right) \end{aligned}$$

where ${S}_{C}$ indicates the number of cases of case category *C*.

### Graph neural networks

**Figure 3:**
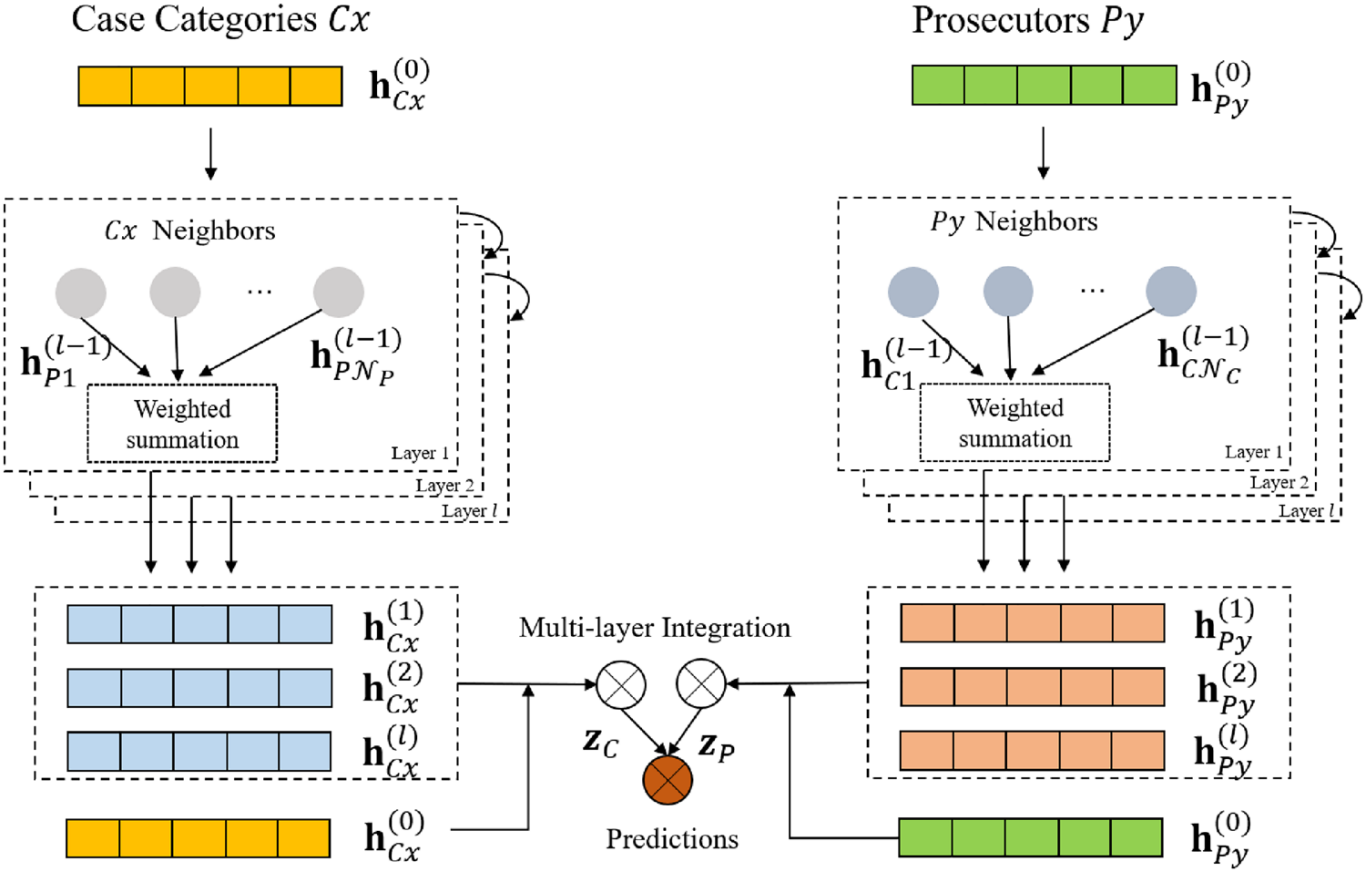
Graph Neural Network Recommendation Model.

Based on the heterogeneous graph *G* constructed above, we design a heterogeneous graph neural model to implement the case assignment. To reduce the complexity of the encoder, we consult the simple structure LightGCN^4^ as the basic structure of the encoder. Figure 3 shows the framework of the recommendation model, which has three main components: the embedding layer, the embedding propagation layer, and the prediction layer. We will describe the whole framework in detail as follow.

#### Embedding layer

In the embedding layer, the input is the learned case category feature vector $\mathcal {X}_{c}$ ( prosecutor ability feature $\mathcal {B}_{p}$ ), which is used as the initial embedding representation $\mathrm {h}_{C} \in \mathbb {R}^{d}\left( \mathrm {h}_{P} \in \mathbb {R}^{d}\right)$, and where $\mathcal {X}_{c}=\frac{1}{S_{C}} \sum _{e \in S_{C}} \mathcal {A}_{e}$. The *d* denotes the size of the embedding representation. The parameter matrix is constructed as an embedding representation index table.

7 $$\begin{aligned} \mathbf {h}^{(o)}=\left\{ \underbrace{\left[ \mathrm {h}_{P 1}^{(o)}, \cdots , \mathrm {h}_{P M}^{(o)}\right] }_{\text{ prosecutor } \text{ embedding } }, \underbrace{\left[ \mathrm {h}_{C 1}^{(o)}, \cdots , \mathrm {h}_{C N}^{(o)}\right] }_{\text{ case } \text{ category } \text{ embedding } }\right\} {\mathop {\longleftarrow }\limits ^{ \text{ Normalization } }} \underbrace{\left\{ \mathcal {B}_{p}, \mathcal {X}_{c}\right\} }_{\text{ PPD,CFS } } \end{aligned}$$

Furthermore, to enrich the relationship between prosecutors and case types, we replaced the frequency of interaction with the learned expected workload. Therefore, the adjacency matrix $D_{p c}$ is converted to the expected workload matrix $W_{p c}$.

8 $$\begin{aligned} W_{p c}=\left[ \begin{array}{ccc} W_{11} &{} \cdots &{} W_{1 N} \\ \vdots &{} \ddots &{} \vdots \\ W_{M 1} &{} \cdots &{} W_{M N} \end{array}\right] {\mathop {\longleftarrow }\limits ^{\mathrm {CWP}}} D_{p c}=\left[ \begin{array}{ccc} d_{11} &{} \cdots &{} d_{1 N} \\ \vdots &{} \ddots &{} \vdots \\ d_{M 1} &{} \cdots &{} d_{M N} \end{array}\right] \end{aligned}$$

#### Embedding propagation layers

The input in the embedding propagation layer is the expected workload matrix of the prosecutor’s case processing. We update the embedding representation of the node at the *k*-th embedding propagation layer based on the higher-order connectivity of the nodes.

9 $$\begin{aligned} \mathbf {h}_{P}^{(k+1)}= & {} \sum _{C \in \mathcal {N}_{P}} \frac{1}{\sqrt{|\mathcal {N}_{P}|} \sqrt{|\mathcal {N}_{C}|}} \mathcal {U}_{P C} \cdot W_{P C} \cdot \mathbf {h}_{C}^{(k)} \end{aligned}$$

10 $$\begin{aligned} \mathbf {h}_{C}^{(k+1)}= & {} \sum _{P \in \mathcal {N}_{C}} \frac{1}{\sqrt{|\mathcal {N}_{C}|} \sqrt{|\mathcal {N}_{P}|}} \mathcal {U}_{C P} \cdot W_{P C} \cdot \mathbf {h}_{P}^{(k)} \end{aligned}$$

At the propagation layer, we adopt symmetric normalization terms $\frac{1}{\sqrt{|\mathcal {N}_{P}|} \sqrt{|\mathcal {N}_{C}|}}$ designed of standard GCN^31^, where the $\mathcal {N}_{P}$ and $\mathcal {N}_{C}$ indicate the number of case categories handled by prosecutor *P* and the number of prosecutors handing case category *C*, respectively. The $\mathcal {U}$ and *W* denote the attention weight and workload of case category *C* handled by prosecutor *P*, respectively. The attention weights will be analyzed later.

#### Prediction layer

We update the embedding representations of nodes by the propagation layers of the *l*-layer. Finally, we aggregate the embedding representations of nodes from different propagation layers.

11 $$\begin{aligned} \mathbf {z}_{P}=\sum _{k=0}^{l} \alpha _{k} \mathbf {h}_{P}^{(k)} ; \mathbf {z}_{C}=\sum _{k=0}^{l} \alpha _{k} \mathbf {h}_{C}^{(k)} \end{aligned}$$

Where $\alpha _{k} \ge 0$ indicates the weighting parameter of the embedding representation of the *k*th layer in the final embedding representation. In this paper, we set $\alpha _{k}$ uniformly to $1 /(K+1)$.

The dot product of the embedding representations of the nodes is used as a prediction function to obtain the scores of the prosecutors for each case category finally.

12 $$\begin{aligned} \hat{y}_{P C}=\mathbf {z}_{P}^{T} \mathbf {z}_{C} \end{aligned}$$

### Interaction attention

**Figure 4:**
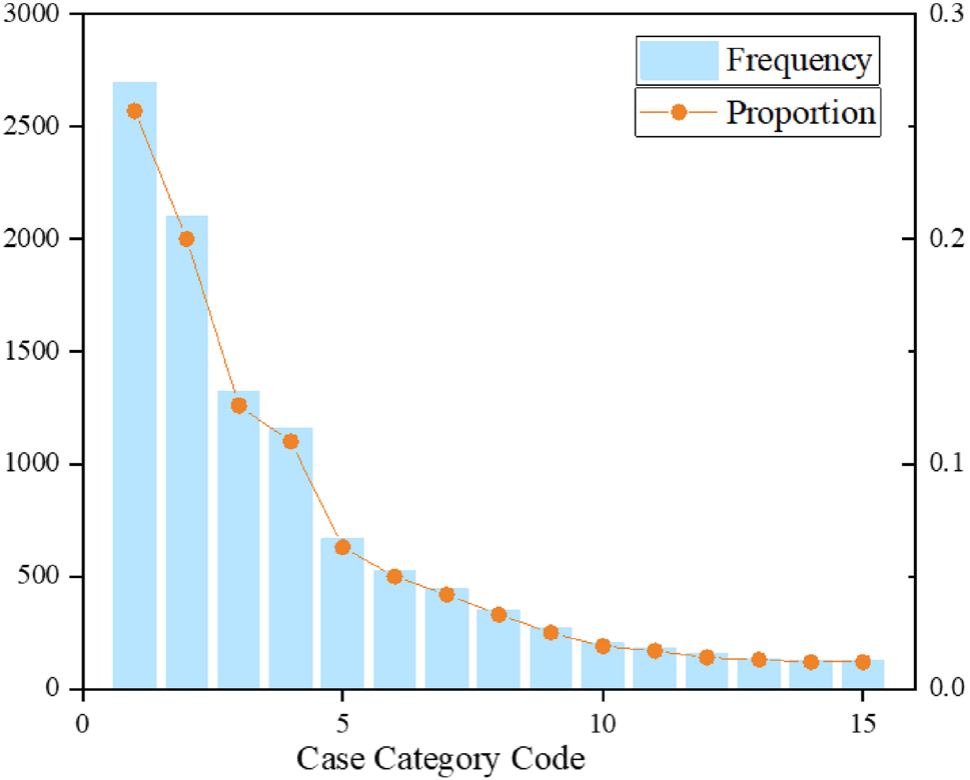
Frequency distribution of the top 15 most frequent codes.

Case assignment usually prioritises prosecutors who have interacted, which inevitably leads to an accumulation of cases. This is the reason that the distributional relationship between prosecutors and case types usually shows a power-law distribution. As shown in Fig. 4, the long-tail part consisting of low case categories lacks supervisory signals. While on the contrary, the high case category frequently appears in the neighbour aggregation and objective function, which has a more significant representational learning influence, making the model biased to recommend head case categories at the expense of long-tail case category exposure. Therefore, We apply an attention mechanism to the interaction to balance low-tailed and high-tailed message learning.

13 $$\begin{aligned} \begin{aligned} \mathcal {U}_{P C}&=\frac{\exp \left( Z_{P C}\right) }{\sum _{j=1}^{\mathcal {N}_{P}} \exp \left( Z_{P j}\right) } \\ \mathcal {U}_{C P}&=\frac{\exp \left( Z_{C P}\right) }{\sum _{j=1}^{\mathcal {N}_{C}} \exp \left( Z_{C j}\right) } \end{aligned} \end{aligned}$$

where $Z_{P C}$,$Z_{C P}$are calculated as

$$\begin{aligned} \begin{aligned}{}&Z_{P C}=MLP\left( \mathbf {h}_{P}^{(k-1)}, \mathbf {h}_{C}^{(k)}\right) \\&Z_{C P}=MLP\left( \mathbf {h}_{C}^{(k-1)}, \mathbf {h}_{P}^{(k)}\right) \end{aligned} \end{aligned}$$

where *MLP*(*x*, *y*) denotes the multilayer perceptron function for the inputs *x* and *y*.

### Model learning

The learning of the model consists of two parts, namely, a pre-training part and a joint learning part. We optimize the self-supervised learning objective in the pre-training part by three different modelling learning. In the joint part, we initialize the encoder with the learned feature and correlation representations and then train the network with supervised learned signals. We use the BPR loss function^1^ to jointly pre-train the loss function to optimize the recommendation model. The BPR loss functions is as follows, which is a pairwise loss that encourages the prediction of an observed entry to be higher than its unobserved counterparts as follows.

14 $$\begin{aligned} \mathcal {L}_{B P R}=-\sum _{P=1}^{M} \sum _{C \in \mathcal {N}_{P}} \sum _{C^{\prime } \notin \mathcal {N}_{P}} \ln \sigma \left( \hat{y}_{P C}-\hat{y}_{P C^{\prime }}\right) +\lambda \Vert E\Vert _{2}^{2} \end{aligned}$$

where $\lambda$ controls the coefficient of L2 regularization. Thus, the overall recommended model loss function is as follows.

15 $$\begin{aligned} \mathcal {L}=\mathcal {L}_{B P R}+\beta _{1} \mathcal {L}_{P P E}+\beta _{2} \mathcal {L}_{C W P}+\beta _{3} \mathcal {L}_{C F S} \end{aligned}$$

Where $\beta _{1}$, $\beta _{2}$, and $\beta _{3}$ denote the hyper-parameters to control the impact effects of the three pre-training tasks, respectively. In this paper, we set three hyperparameters to 1, 0.3 and 0.5 with experimental analysis, respectively.

## Experiment

We conducted experiments on real-world data sets to prove the method’s effectiveness in this paper. We also provided multiple baseline methods for comparative analysis to confirm the method’s robustness.

### Experimental settings

#### Data description and preprocessing

We collected the dataset from the case handling data of the three procuratorates in China. Table 1 shows the situation of the dataset. We obtained the following concepts from the historical case-handling set containing.

- *Prosecutor data sets* Prosecutor data are formatted data, including the identity features and case handling features of prosecutors, such as the gender, age, number of cases, and number of cases categories handled. Those features assist in portraying the competency profile of prosecutors.
- *Case category data sets* A case category set is a set of all cases categories handled by prosecutors. For example, “Robbery, Fraud”.
- *Case data sets* This set includes all cases handled by prosecutors during the period. These data provide support for case feature selection.

We performed the following pre-processing steps to use the multi-source heterogeneous case dataset fully. First, for the prosecutor data set, we counted the natural and case features of prosecutors from the historical case and the biological database. Second, for the case category data set, we counted the case categories handled by each prosecutor from the historical case database and encode all the case categories with labels. Finally, for the case data set, we extracted case data from the historical case database. In addition, we filter out prosecutors and case categories with less than five interaction records.

**Table 1:** Statistics of our dataset.

Description	Number
The number of prosecutors	106
The number of case categories	146
The number of cases	25175
The number of case features	78
The number of prosecutor features	32
The average workload of prosecutors handling cases	5.4
The average number of cases handled by prosecutors	238
The average number of case categories handled by prosecutors	22

### Evaluation metrics

We generate a ranked list of the top *K* cases from the output and set the *K* distribution to 5 and 10. we use precision, recall, personal hit rate (PHR {5,10}) and normalized discounted cumulative gain (NDCG{5,10}) as evaluation metrics.

Precision and recall are the standard metrics to evaluate model performance. For prosecutor $p_{i}$, the precision and recall rate is calculated as follows.

16 $$\begin{aligned} Precision\left( p_{i}\right)= & {} \frac{|\hat{S}_{i} \cap S_{i}|}{|\hat{S}_{i}|} \end{aligned}$$

17 $$\begin{aligned} Recall\left( p_{i}\right)= & {} \frac{|\hat{S}_{i} \cap S_{i}|}{|S_{i}|} \end{aligned}$$

Where $\hat{S}_{i}$,$S_{i}$ denote the predicted and actual Top-K results of prosecutor $p_{i}$, respectively. The $|S_{i}|$ denotes the set size of prosecutor $p_{i}$.

PHR is a user-level based evaluation metric that indicates the proportion of users whose predicted set contains true elements. For prosecutor $p_{i}$, PHR is calculated as follows.

18 $$\begin{aligned} {\text {PHR@K}}\left( p_{i}\right) =\frac{\sum _{i=1}^{N^{\prime }} \varphi \left( |\hat{S}_{i} \cap S_{i}|\right) }{N^{\prime }} \end{aligned}$$

where $N^{\prime }$ denotes the number of prosecutors in the test set, and $$\varphi \left( |\hat{S}_{i} \cap S_{i}|\right) =\left\{ \begin{array}{cc}1 &{} |\hat{S}_{i} \cap S_{i}|\ge 0 \\ 0 &{} \text{ otherwise } \end{array}\right.$$.

NDCG is a measure of ranking quality that considers the order of the elements in the recommendation list. For prosecutor $p_{i}$, the NDCG is calculated as follows.

19 $$\begin{aligned} {\text {NDCG@K}}\left( p_{i}\right) =\frac{\sum _{k=1}^{K} \frac{\delta \left( \hat{S}_{i}^{k}, S_{i}\right) }{\log _{2}(k+1)}}{\sum _{k=1}^{\min \left( K,|S_{i}|\right) } \frac{1}{\log _{2}(k+1)}} \end{aligned}$$

Where $$\delta (v, S)= {\left\{ \begin{array}{ll}1 &{} v \in S \\ 0 &{} v \notin S\end{array}\right. }$$.

### Baselines

We compare our proposed method with the following four methods.

- *MF*^1^ This is a matrix decomposition of Bayesian personalized ranking (BPR) loss optimization, which utilizes only the direct user-item interaction as the objective value of the interaction function.
- *NeuMF*^13^ The approach is a state-of-the-art neural CF model that uses multiple hidden layers on top of the user and item embedded elements and links to capture their nonlinear feature interactions. In particular, we use a two-layer planar structure where the dimensionality of each hidden layer is kept constant.
- *LightGCN*^4^ A general recommendation model based on GCN, which uses user-item proximity to learn node representations and generate recommendations, is reported to be state-of-the-art.
- *SGL*^26^ This is a state-of-the-art self-supervision graph neural network recommendation method. The idea of the algorithm is to alter the original graph data with uniformly missing points/edges discarded to generate new views of the data, and then learn the generalization ability based on the comparison learning of different views.

Notably, most existing recommendation models mainly focus on social or product recommendations. To compare the performance of existing methods and our method in the judicial domain, we compare the traditional collaborative filtering MF, NCF, and the state-of-the-art LightGCN model. In addition, to verify the effectiveness of the self-supervised learning strategy, we compare our method with the existing self-supervised learning model GSL.

### Experimental parameter setting

We divide the dataset into the training set, validation set and test set in the ratio of 70%, 10% and 20% for experiments. After data fixation, we train our model with a fixed number of epochs (1000 epochs) and obtain the best performance of the model on the validation set for testing. However, to speed up the model convergence and reduce the loss function to the lowest point, we set different learning rates, 0.001, 0.0005, and 0.00001, for model training. Moreover, the corresponding batch size is set to 32, 64 and 64 when we modify the learning rate. We construct the model based on LightGCN^4^ and Adam^32^ is used as the optimizer. We set the dimension *k* of hidden factors to 64 and the batch size to 1000. Last, we choose the model that achieves the best test set performance. To evaluate the method’s performance accurately, we generate a ranking list of top-K elements from the output, where *K* is 5 and 10, respectively. Furthermore, to avoid gradient vanishing in the learning process of the network, we set the number of network layers to 2.

## Analysis of experimental results

In this section, we first analyze the overall experimental result comparison in general, then examine each pre-training generative task compared to traditional methods. Finally, we also perform ablation experiments to explore the improvement effect of each task on recommendation performance. In addition, we analyze the effects of network sparsity and hyper-parameters on model performance.

### Overall performance comparison

In this section, we verify whether our method can improve the rationality of case assignment. A comparison of the baseline model results and techniques in this paper is shown in Table 2. From the results, we can get the following experimental analyses.

**Table 2:** The performance of each model.

Model	Precision	Recall	PHR@5	PHR@10	NDCG@5	NDCG@10
MF	0.3732	0.5176	0.5035	0.5267	0.4428	0.4939
NeuMF	0.3843	**0.5381**	0.5121	0.5237	0.5198	0.5206
LightGCN	0.4864	0.5284	0.5442	0.5538	0.5237	**0.5521**
SGL	**0.5154**	0.5368	**0.5543**	**0.5962**	**0.5278**	0.5437
SGPGRec	*0.5182*	*0.5493*	*0.5652*	*0.5924*	*0.5303*	*0.5546*

The best results in the comparison methods are in bold, and the results of our method are in italic.

First, our method performed significantly better than the benchmark models. The experimental results proved that the proposed technique could effectively fuse the increased prosecutor and case category feature representations and the correlation between them into the graph neural network recommendation model to achieve a reasonable case assignment. Our model can obtain better recommendation performance and further demonstrates that constructing self-supervised signals with node representations and node relationships can help the model capture suitable case assignment patterns.

Second, for the baseline approach of traditional MF, we considered that MF and NeuMF perform were worse than LightGCN and SGL on different metrics. LightGCN and SGL used graph neural networks to capture the higher-order connectivity of nodes and mine potential matching possibility. These results validated that graph neural networks were more suitable for rational case assignment than traditional collaborative filtering methods.

Third, the LightGCN and SGL had the same graph neural network structure, but the input of the graph is different. LightGCN modelled the higher-order connectivity of nodes with graph convolutional neural networks to predict items. SGL added some self-supervised learning tasks to generate the graph structure data based on the original LightGCN. The results showed that SGL consistently outperformed the LightGCN model, which indicated the importance of enhancing graph structure with self-supervised tasks.

Finally, for the same LightGCN model based on the self-supervised learning task, the performance of our method was superior to SGL on the actual data set. The three self-supervised learning tasks of SGL were mainly to alter the original data graph to generate new data views to learn the generalized representation. This self-supervised approach with different views was from the graph structure, and the generated supervised signals had some limitations. In contrast, our method built three pre-training tasks to create the feature representation of nodes and the correlation between nodes from the actual data. Therefore, our approach was significantly better than the SGL method.

### The performance of prosecutor portrait depiction

Since our model aims to improve the recommendation performance with pre-training task, we validate whether our method can achieve accurate prosecutor modelling. We adopted the regression task to portray the features of prosecutors in five dimensions, including natural information, case quantity, case quality, case efficiency, and case effectiveness, respectively. They were from three characteristic indicator systems: quantitative, proportional, and distributional. In the comparison experiments, we used the traditional linear regression model as the baseline comparison method to compare the performance of prosecutors’ modelling.

**Figure 5:**
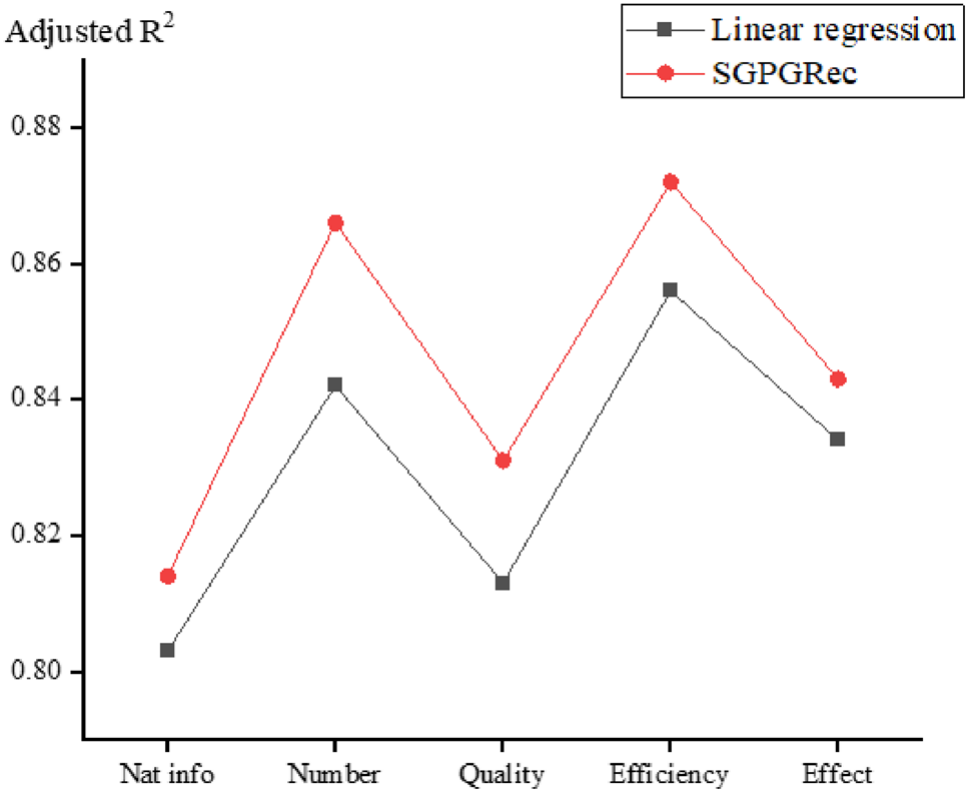
The performance of prosecutor portrait engraving.

The experimental results are shown in Fig. 5, and we used the adjusted R-square as the evaluation metric. As seen from the table, the model’s performance in this paper was significantly improved in modelling prosecutors. The experimental results proved that our model could learn the practical prosecutor features and thus improve the recommendation performance.

### The performance of case workload prediction

We adopted a workload prediction task to generate the expected workload for prosecutors’ cases. To verify whether the expected workload generated by our method could represent the interaction between prosecutors and case categories better, we evaluated our proposed model with different methods. Specifically, we selected ten prosecutors to handle Top-20 case categories for experimental comparison. We compared our model with the actual case workload: (1) The expected case workload is statistically derived from the professionals analysis. (2) Decision regression tree: the expected case workload was predicted by a decision tree and trained with the losses described in Eq. (3).

**Figure 6:**
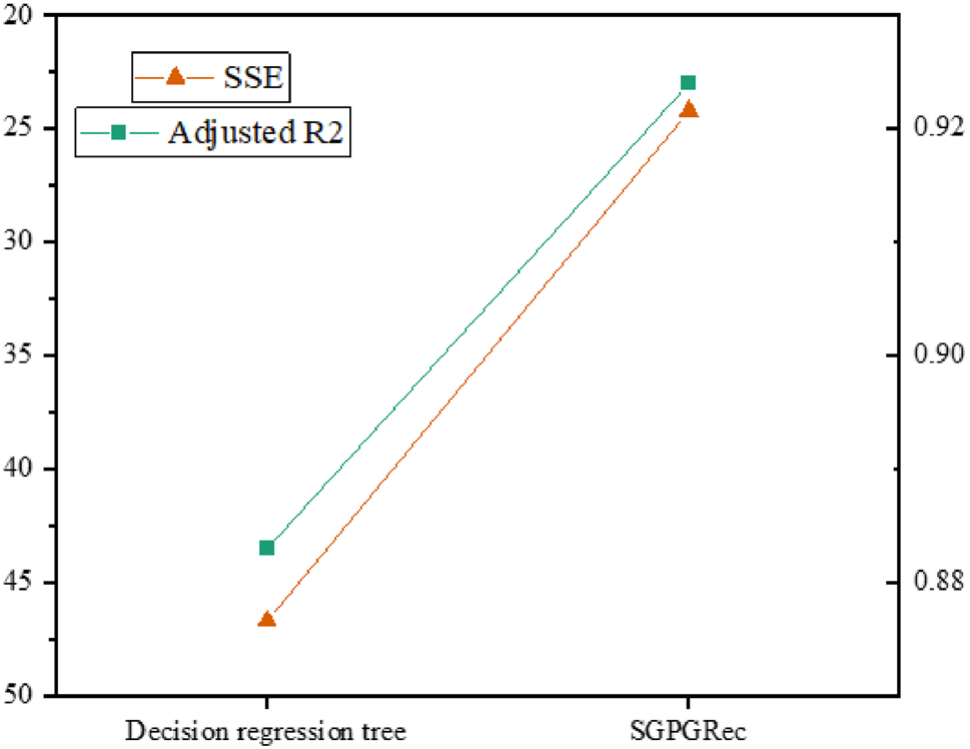
The performance of case workload prediction.

The results are shown in Fig. 6. We adopted the adjusted $R^{2}$ and the sum of squares due to error (SSE) as the evaluation metrics. From the test results, the performance of our method was significantly better than the benchmark model and close to the actual case expectation workload. The results showed that the model could effectively predict the expected workload of cases and realize the modelling of interaction information, thus improving the recommendation performance.

### The performance of case features selection

In this paper, we employed a classification task to select the features that affect the case complexity. To verify whether this method is good at selecting features, we adopted our method and the traditional method for experimental comparison. In particular, we first selected Top-20 features by the traditional method and our method and then invited professionals from the prosecutor’s office to evaluate and score the features selected by both methods and calculated the feature selection score.

**Figure 7:**
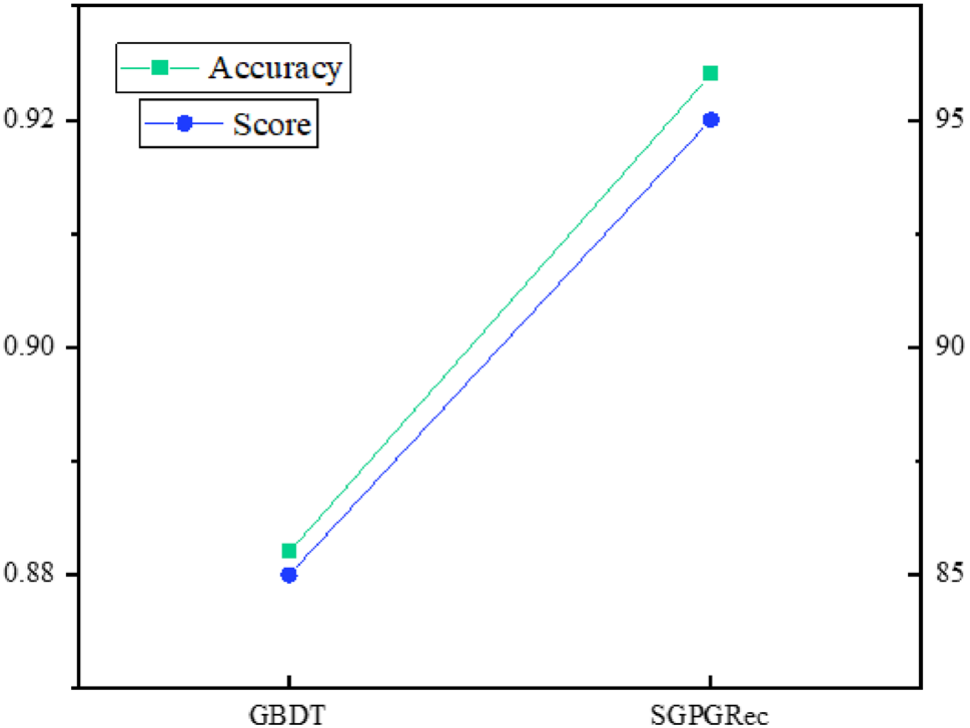
The performance of case features selection.

The results are shown in Fig. 7. We employed classification accuracy and feature selection scores as evaluation metrics. From the test results, the performance of our feature selection method was significantly better than the benchmark model. The results show that the model can effectively select features that affect case complexity, thus improving the performance of the recommended model.

### Ablation study

To explore the impact of the three pre-training tasks on the recommended performance, we conducted an ablation experiment, and the results are shown in Table 3. To be specific, we offered the scores when the different training tasks were turned off. Here, h/n PPD, h/n CWP,h/n CFS, and h/n ALL referred to pre-training the model without prosecutor portrait engraving, case workload prediction, case feature selection, and none of them, respectively. We also provided the results of the baseline LightGCN with the best overall performance for comparison.

**Table 3:** Comparison of the impact on recommendation performance for different tasks.

Metrics	Precision	Recall	PHR@5	PHR@10	NDCG@5	NDCG@10
Light GCN	0.4864	0.5284	0.5442	0.5538	0.5237	0.5521
h/n PPD	0.4996	0.5332	0.5490	0.5563	0.5258	0.5528
h/n CWP	**0.5124**	**0.5435**	**0.5589**	**0.5764**	**0.5294**	**0.5539**
h/n CFS	0.5075	0.5387	0.5456	0.5688	0.5288	0.5532
h/n ALL	0.4921	0.5294	0.5446	0.5544	0.5246	0.5525
SGPGRec	*0.5182*	*0.5493*	*0.5652*	*0.5924*	*0.5303*	*0.5546*

The best results in ablation experiments are in bold, and the results of complete method are in italic.

The results showed that all three pre-training tasks contributed to the primary model, as the absence of any tasks led to decreased performance. Notably, the model also outperformed LightGCN in the lack of the self-supervised study, indicating that the attention strategy can also improve the model’s performance. Furthermore, the model with case feature selection (h/n CWP) or case workload prediction (h/n CFS) yielded better results compared to the pre-trained model without prosecutor portrait depiction (h/n PPD). It further demonstrated that the self-supervised tasks could help the models capture the higher-order connectivity in the interactions and inject key representations into the pre-trained models.

It was worth noticing that the model only with two pre-training tasks (h/n CWP) could achieve comparable performance in terms of PHR. It demonstrated that these two pre-training tasks had similar generated feature representations.

The dataset contained a heterogeneous graph with many cases and prosecutor features. We proposed that three pre-training tasks with generated feature representation and interaction could significantly improve the model performance and validate the method’s effectiveness and robustness.

### Performance on sparse network

**Figure 8:**
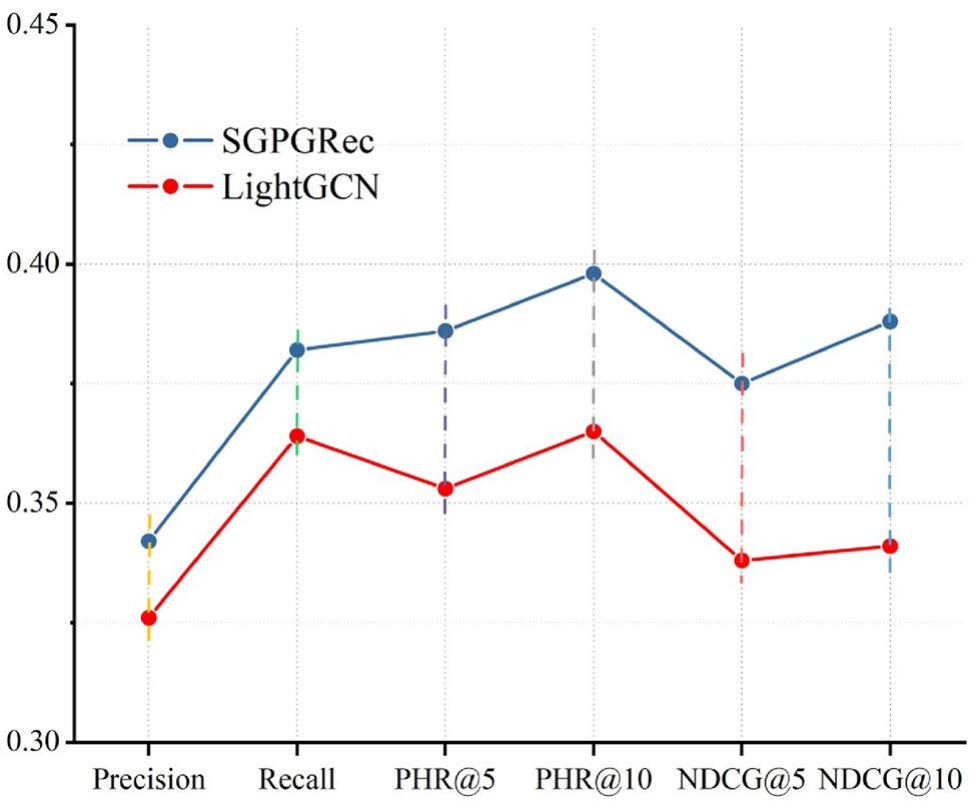
Performance w.r.t.Network Sparsity.

As shown in Fig. 4, the relationship between prosecutors and case categories is a power-law distribution. To further explain how the model performs when the network was sparse, we conducted an experimental analysis with the tail data of the power-law distribution. We selected the case categories with less than 500 interactions for the experiment, which accounted for about 20% of the total data. Furthermore, we also chose LightGCN as the benchmark method for comparison experiments. The experimental results are shown in Fig. 8.

As can be seen from Fig. 8, the SGPGRec still outperformed the LightGCN model significantly in different evaluation metrics when the network was sparse. It further demonstrated that the proposed model can capture not only the head signals but also the tail signals in power-law distribution data.

### Hyper-parameters studies

When applying SGPGRec to the actual dataset, besides the L2 regularization coefficient $\lambda$ following the LightGCN, the most important hyper-parameters to tune are for the loss functions of the three pre-training tasks. Since the essential part of the loss function is the BPR loss function, the weights $\beta _{1}$, $\beta _{2}$, $\beta _{3}$ of the loss function for the three pre-training tasks are less than or equal to 1.

Hence, we chose values of 0.3, 0.5, and 1 for each hyper-parameter, and then tested the effect of each hyper-parameter on the experimental results with the controlled variable method, respectively. When we adjusted one of the hyper-parameters, the other two hyper-parameters were kept equal to 0.5. Here, we selected the recommendation precision and recall as the evaluation metrics. The effect of hyper-parameters on model performance is shown in the following figure.

**Figure 9:**
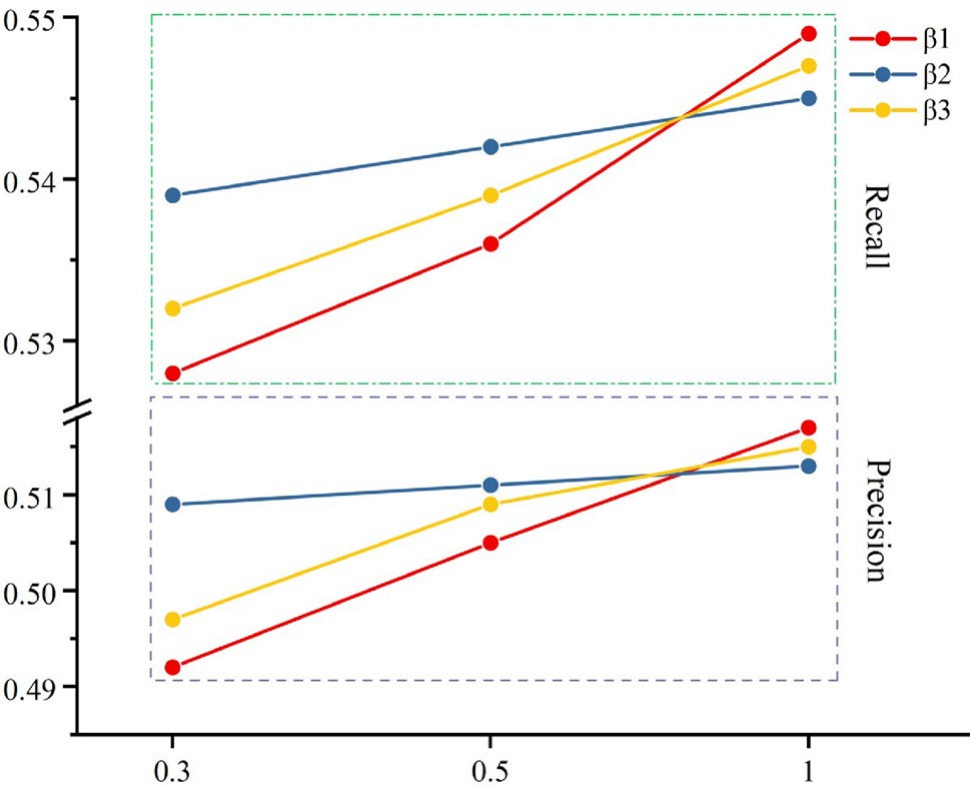
Performance w.r.t. different hyper-parameters $\beta _{1}$, $\beta _{2}$, $\beta _{3}$.

From the Figure 9, the changes in precision and recall were more obvious when we adjusted $\beta _{1}$, which indicated that PPE had the greatest impact on the overall model. Moreover, when we adjusted $\beta _{2}$, the changes in precision and recall were minimal, which indicated that CWP has the least impact on the overall model. Therefore, In the pre-training stage, we set the hyper-parameters for three losses (i.e., PPE, CWP, CFS) as $\beta _{1}=1$, $\beta _{2}=0.3$, and $\beta _{3}=0.5$, respectively.

### Application

**Figure 10:**
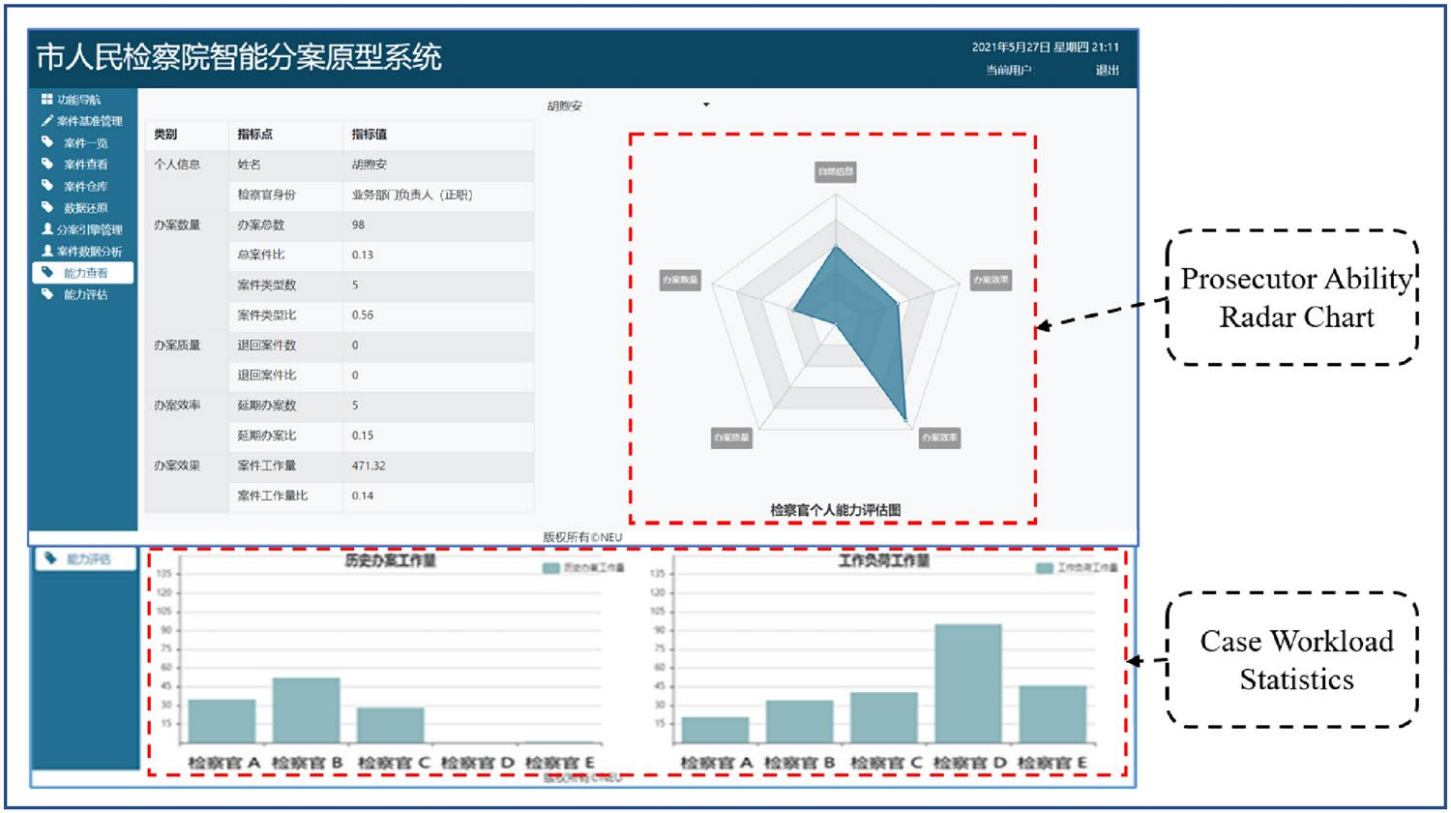
The application of SGPGRec.

**Figure 11:**
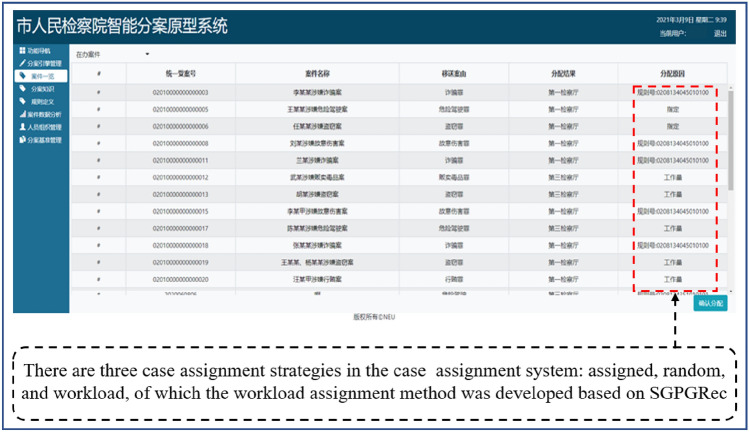
Visualization of pre-training generative tasks results.

We applied successfully the SGPGRec technologies to a procuratorate’s intelligent case assignment system in China. As shown in Fig. 10, when we input cases, the system will recommend suitable prosecutors. Moreover, we visualized the results of different pre-training tasks in SGPGRec, and the results were shown in Fig. 11.

## Conclusion

This paper studies the prosecutor case matching problem and proposes an end-to-end graph neural network recommendation model based on self-supervised learning. To be specific, we design three auxiliary self-supervised tasks based on the prosecutor-case category interaction graph and the data distribution to obtain the feature representations of prosecutors, case categories, and the interaction between them. Then an end-to-end graph neural network recommendation model is constructed using the interaction attention mechanism based on the data with the power-law distribution. Extensive experimental consistency on real datasets from three procuratorates shows that our approach is practical compared to several competing baseline approaches and further supports developing an intelligent case assignment system with adequate performance.

## Data Availability

The data used in this paper are from the real case data of the prosecutor’s office, and so it cannot be made freely available. Requests for access to these data should be made to the corresponding author.

## References

[1] Rendle, S.: Bpr: Bayesian personalized ranking from implicit feedback. arXiv preprint arXiv:1205.2618 486, 75–174 (2012).

[2] He X (2018). Nais: Neural attentive item similarity model for recommendation. IEEE Trans. Knowl. Data Eng..

[3] Koren, Y.: Factorization meets the neighborhood: A multifaceted collaborative filtering model. In: Proceedings of the 14th ACM SIGKDD International Conference on Knowledge Discovery and Data Mining, pp. 426–434 (2008).

[4] He, X., Deng, K: Lightgcn: Simplifying and powering graph convolution network for recommendation. In: Proceedings of the 43rd International ACM SIGIR Conference on Research and Development in Information Retrieval, pp. 639–648 (2020).

[5] Berg, R., Kipf, T.N., Welling, M.: Graph convolutional matrix completion. arXiv preprint arXiv:1706.02263 (2017).

[6] Wu, S., Sun, F., Zhang, W., Xie, X., Cui, B.: Graph neural networks in recommender systems: A survey. ACM Computing Surveys (CSUR) (2020).

[7] Zare, A., Motadel, M.R., Jalali, A.: A hybrid recommendation system based on the supply chain in social networks. *J. Web Eng.* pp 633–660 (2022).

[8] Hamilton, W.L.: Inductive representation learning on large graphs. In: Proceedings of the 31st International Conference on Neural Information Processing Systems, pp. 1025–1035 (2017).

[9] Wu Z, Pan S, Chen F, Long G, Zhang C, Philip SY (2020). A comprehensive survey on graph neural networks. IEEE Trans. Neural Netw. Learn. Syst..

[10] Gao, C., Zheng, Y.: Graph neural networks for recommender systems: Challenges, methods, and directions. arXiv preprint arXiv:2109.12843 (2021).

[11] Wang, X., He, X.: Neural graph collaborative filtering. In: Proceedings of the 42nd International ACM SIGIR Conference on Research and Development in Information Retrieval, pp. 165–174 (2019).

[12] Kipf, T.N., Welling, M.: Semi-supervised classification with graph convolutional networks. arXiv preprint arXiv:1609.02907 (2016).

[13] He, X., Liao, L.: Neural collaborative filtering. In: Proceedings of the 26th International Conference on World Wide Web, pp. 173–182 (2017).

[14] Liu, X., Zhang, F., Hou, Z.: Self-supervised learning: Generative or contrastive. IEEE Transactions on Knowledge and Data Engineering (2021).

[15] Gidaris, S.: Unsupervised representation learning by predicting image rotations. arXiv preprint arXiv:1803.07728 (2018).

[16] Oord, A., Li, Y., Vinyals, O.: Representation learning with contrastive predictive coding. arXiv preprint arXiv:1807.03748 (2018).

[17] Devlin, J.: Bert: Pre-training of deep bidirectional transformers for language understanding. arXiv preprint arXiv:1810.04805 (2018).

[18] Lan, Z., Chen, M.: Albert: A lite bert for self-supervised learning of language representations. arXiv preprint arXiv:1909.11942 (2019).

[19] Peng, Z., Huang, W., Luo, M., Zheng, Q., Rong, Y., Xu, T., Huang, J.: Graph representation learning via graphical mutual information maximization. In: Proceedings of the Web Conference 2020, pp. 259–270 (2020).

[20] Qiu, J., Chen, Q., Dong, Y.: Gcc: Graph contrastive coding for graph neural network pre-training. In: Proceedings of the 26th ACM SIGKDD International Conference on Knowledge Discovery & Data Mining, pp. 1150–1160 (2020).

[21] Sun, K., Lin, Z., Zhu, Z.: Multi-stage self-supervised learning for graph convolutional networks on graphs with few labeled nodes. In: Proceedings of the AAAI Conference on Artificial Intelligence, vol. 34, pp. 5892–5899 (2020).

[22] Sun, F.-Y.e.a.: Infograph: Unsupervised and semi-supervised graph-level representation learning via mutual information maximization. arXiv preprint arXiv:1908.01000 (2019).

[23] Velickovic P (2019). Fedus.: Deep graph infomax. ICLR (Poster).

[24] Ma, J., Zhou, C.: Disentangled self-supervision in sequential recommenders. In: Proceedings of the 26th ACM SIGKDD International Conference on Knowledge Discovery & Data Mining, pp. 483–491 (2020).

[25] Zhou, K., Wang, H.: S3-rec: Self-supervised learning for sequential recommendation with mutual information maximization. In: Proceedings of the 29th ACM International Conference on Information & Knowledge Management, pp. 1893–1902 (2020).

[26] Wu, J., Wang, X.: Self-supervised graph learning for recommendation. In: Proceedings of the 44th International ACM SIGIR Conference on Research and Development in Information Retrieval, pp. 726–735 (2021).

[27] Xiao, C., Xie, R., Yao, Y.: Uprec: User-aware pre-training for recommender systems. arXiv preprint arXiv:2102.10989 (2021).

[28] Yu, J., Yin, H., Gao, M.: Socially-aware self-supervised tri-training for recommendation. arXiv preprint arXiv:2106.03569 (2021).

[29] Yao, T., Yi, X.: Self-supervised learning for deep models in recommendations. arXiv e-prints, 2007 (2020).

[30] Ke, G., Xu, Z., Zhang, J.: Deepgbm: A deep learning framework distilled by gbdt for online prediction tasks. In: Proceedings of the 25th ACM SIGKDD International Conference on Knowledge Discovery & Data Mining, pp. 384–394 (2019).

[31] Welling, M., Kipf, T.N.: Semi-supervised classification with graph convolutional networks. In: J. International Conference on Learning Representations (ICLR 2017) (2016).

[32] Kingma, D.P., Ba, J.: Adam: A method for stochastic optimization. arXiv preprint arXiv:1412.6980 (2014).

